# Search extension transforms Wiki into a relational system: A case for flavonoid metabolite database

**DOI:** 10.1186/1756-0381-1-7

**Published:** 2008-09-17

**Authors:** Masanori Arita, Kazuhiro Suwa

**Affiliations:** 1 Department of Computational Biology, Graduate School of Frontier Sciences, The University of Tokyo, Kashiwanoha 5-1-5 CB05, Kashiwa, Japan; 2 Metabolome Informatics Unit, Plant Science Center, RIKEN, Japan; 3 Institute for Advanced Biosciences, Keio University, Japan

## Abstract

**Background:**

In computer science, database systems are based on the relational model founded by Edgar Codd in 1970. On the other hand, in the area of biology the word 'database' often refers to loosely formatted, very large text files. Although such bio-databases may describe conflicts or ambiguities (e.g. a protein pair do and do not interact, or unknown parameters) in a positive sense, the flexibility of the data format sacrifices a systematic query mechanism equivalent to the widely used SQL.

**Results:**

To overcome this disadvantage, we propose embeddable string-search commands on a Wiki-based system and designed a half-formatted database. As proof of principle, a database of flavonoid with 6902 molecular structures from over 1687 plant species was implemented on MediaWiki, the background system of Wikipedia. Registered users can describe any information in an arbitrary format. Structured part is subject to text-string searches to realize relational operations. The system was written in PHP language as the extension of MediaWiki. All modifications are open-source and publicly available.

**Conclusion:**

This scheme benefits from both the free-formatted Wiki style and the concise and structured relational-database style. MediaWiki supports multi-user environments for document management, and the cost for database maintenance is alleviated.

## Background

### Why is database maintenance unappreciated?

In many research fields, building or maintaining a database system is not a sought-after task and researchers tend to avoid the chore because: 1) the inputting and checking of data are routine and tedious, 2) novel findings are rarely based on a collection of old data, 3) database developers often do not receive deserved credit especially when data are distributed for free, and 4) it is difficult to evaluate the quality and value of data. However, most bioinformatics research requires high-quality databases. Their significance is clear from the success of major data-servicing institutes such as the National Center for Biotechnology Information (NCBI; USA) or the European Bioinformatics Institute (EBI; UK). Without doubt, data collection and management are important activities in scientific research.

### Input, management, and query are the keys

How can we promote the development and maintenance of high-quality databases? The key processes in data handling are the input, management, and query of data. The effort required for the first two activities can be significantly reduced by introducing a Wiki-based system. The Wikipedia, a web-based free encyclopaedia, for example, continues its rapid growth despite criticism by experts of 'lack of quality control' and 'vulnerability to website vandals' [1]. Its English version now boasts over 2 million articles followed by 0.7 and 0.6 million in German and French [2]. Still unsupported is flexibility in query mechanisms and presentation such as displaying customized statistics in a user-friendly way.

In the rapidly evolving frontiers of biology research, the development of flexible and systematic query mechanisms has not been pursued actively. Biological data and their formats often co-evolve; the definition of data type in a repository requires frequent, major updates. This is not compatible with the relational model proposed by E. Codd [3]. It has been the gold standard for data management for decades but the model requires fixation of database schema prior to data input. Currently, many biologists prefer using a spreadsheet such as Excel (Microsoft Corp., Seattle, WA, USA) or a simple text to organize their experimental results, and biology databases only provide full-text searches to access large-scale, unstructured text data. Ideally, a bio-database needs to serve as a searchable, digital laboratory notebook where users can efficiently organize and query data. As most Wiki systems only provide a collection of independent pages with weak query functions, we tested the possibility of installing a flexible query mechanism on a Wiki-based system. Here we propose an extension of MediaWiki that can emulate traditional database operations [4]. We demonstrate our idea with the molecular information on flavonoid, the major category of plant secondary metabolites.

This paper is organized as follows. The basic function and flexibility of MediaWiki are introduced in Methods section from a computer-science perspective. Using functionality, we introduce the implementation of the flavonoid database in Results section. The advantages of our approach and differences from other approaches are reviewed in Discussions. Readers are encouraged to access our website at .

## Methods

### Flavonoid data source

Flavonoid is a class of plant secondary metabolites with a C6-C3-C6 skeleton derived from the phenylpropanoid-acetate pathway [5]. This class serves not only as pigments but also as antioxidants, and as anti-inflammatory or anti-cancer agents. Under the name of polyphenols it has drawn much commercial attention [5]. We previously input and classified their molecular structures identified in various plant species [6,7]. Our structural classification assigns a 12-digit alpha-numeral to each structure and can reveal physico-chemical properties, modification patterns, and biosynthetic pathways from an evolutionary perspective. However, the efficient management of these data has remained a goal. As of September, 2008, the data consisted of 6902 flavonoid structures, 1687 plant species, and 4970 primary journal references reporting 19,788 metabolite-species relationships.

### Basic functionality of MediaWiki: extension, template, and namespace

In most Contents Management Systems, page contents are stored in a file system and their edit history is automatically maintained to manage version differences or to restore past edits. MediaWiki excels such systems in the following four characteristics. First, the entire system is built on a Relational Database (RDB). Each article, called a *page*, is separated into its title and body and stored in a single table in the background RDB (in our case, mySQL). Second, MediaWiki supports user-defined functions called *extension*. A system administrator can add extension programs written in PHP language and enhance MediaWiki functionality. By overwriting the source code, any modification can be applied through this option. For example, the **if-then **control command of a programming language has been implemented in MediaWiki as an extension [8]. Third, MediaWiki supports a term-rewriting system called *template*. The template mechanism itself is implemented as a special page that can be pasted into any other page by calling its page title. A template page can accept arguments; its function is similar to a function call of programming languages. Lastly, MediaWiki provides a classification scheme for pages called *namespace*. Each page belongs to a certain namespace which is displayed as a prefix of the page title. In the background RDB, each namespace is translated into an integer value assigned to each data tuple in the relation table. One major custom namespace is *Category*. Pages in this space are used to describe the semantic hierarchy, and all pages linking to a Category page are automatically sorted for display in the page. These functions are fully exercised in our database design. Hereafter, the words *extension, template*, and *namespace *carry the meaning described above.

### Implemented functions to realize relational queries

Text data managed by Wiki-based systems are inherently free-formatted. The fundamental operation for realizing any query is the full-text search. In our implementation, for efficiency, information for systematic searches must be described in tabular form with a special separator, "&&", at the head of each table element. Tabular data are searched and organized with the following new commands implemented as an extension on MediaWiki.

• {{#SearchTitle:arg1|arg2}}: The command lists all page titles that match the regular expression arg1 in namespace arg2. The returned titles are separated by &&.

• {{#SearchLine:arg1|arg2|arg3}}: The command lists all page lines that match the regular expression arg1 in the namespace arg2. An optional argument arg3 specifies a particular page title in the namespace. A page title is attached at the head of every detected line with a separator &&.

• {{#repeat:arg1|arg2|arg3}} The command repeats the application of the template titled arg1 that requires arg2 arguments (arg2 must be a positive integer) for the list of arguments arg3 separated by &&.

These commands are used to emulate two fundamental operators in the relational model: selection (extraction of rows from a relation table) and projection (extraction of columns from a relation table). Note that search results are labeled with page titles, which serve as the identifier of the relation table being searched. A join operation (merging two relation tables by associating a particular column common to both) is theoretically possible by repeating searches for all table elements; however, the computational cost is too high. In our implementation, such costly operations, including set operations, are delegated to the Lua embeddable programming language [9]. Lua is called by a special command #lua:arg1|arg2}}, where arg1 is the program body and arg2 is the input value passed to the standard input of the Lua interpreter. Lua process is invoked by visiting a page; its standard output is embedded where the command is written.

All newly implemented Wiki commands (around 40; most are the functions for string operations) are summarized on our website [10]. Their source codes are also available to reproduce our implementation on MediaWiki.

### Data serialization: inputs and outputs

The page contents of the database can be converted to XML data in MediaWiki; the initial construction and major modification of the contents, including data integration from multiple data sources, were operated in the XML format. In our initial database construction, a sample page for a single flavonoid compound was created and verified its XML format by serializing the page. Then other data were reformatted in the same XML style and were bulk input accordingly. Page update is possible either through a web browser or the OpenOffice Project, which recently announced the function to export Microsoft Office documents to MediaWiki [11].

## Results

### Information tables for compounds, plant species, and references

**Figure 1:**
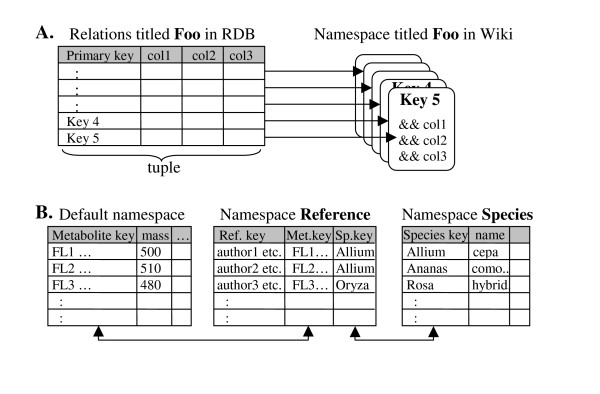
**A) Correspondence between relation table in RDB and pages in MediaWiki;**. One tuple corresponds to one page whose title is the primary key. Together, pages in the same namespace constitute the original relation table. In Wiki pages, attributes are separated by user-defined separator symbols. In this example, && is the separator. B) Basic schema of flavonoid database; A table for reference information connects tables for metabolite and plant species. The link is used for Metabolite-Species table in Wiki pages.

Our basic concept is to describe tabulated data in Wiki pages and to let all other operations such as formatting for visualization, obtaining statistics, or applying relational operators be done by the template mechanism and text-string searches. Information on pages is intended to be least redundant to maintain data integrity and to reduce input and update tasks. The translation scheme between the relational model and Wiki pages is shown in Figure 1A. Each relation table is represented by a single namespace and each data tuple (or a table-row) corresponds to one Wiki page. The separation by namespaces rather than page titles is superior for simpler page classification and management.

Different from traditional metabolite databases (e.g. Merck Index, Chemical Abstract Service), we prepared three relation tables that provide compounds, plant species, and references on an equal footing rather than assigning a compound ID as the sole primary key for all information (Figure 1B). The three-legged structure is derived from a focus on the metabolite-species relationship reported in literatures. Since a single reference may describe multiple metabolite-species relationships, the same literature information may be referred from multiple pages. To minimize redundancy, literature information is recorded only once in the *reference *namespace and necessary information is dynamically integrated by using string-search commands in the *compound *and *species *namespaces.

For example, a page for a compound displays information on plant species and references that is derived automatically from other pages or external resources. Using the 12-digit ID, each compound page is associated with a MOL-format (molecular structure) file from which structural information such as formula, exact mass, and average mass is obtained. The same is true for the *species *pages. The page title is a genus name from which the taxonomic hierarchy is derived. Such derivation is performed either by templates or by the embedded Lua programming language.

#### Case example: (+)-catechin

**Figure 2:**
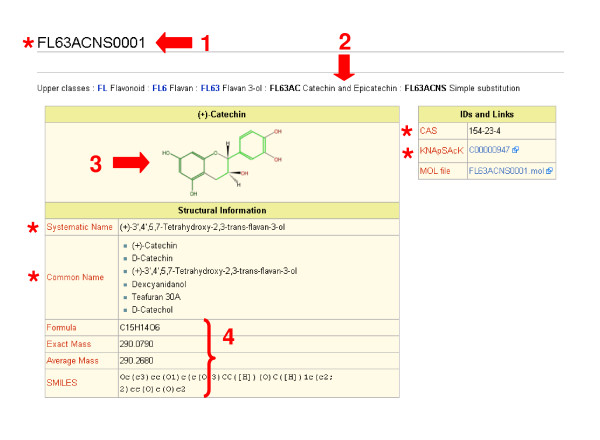
**The molecule view of (+)-catechin;**. Red stars indicate information written on the Wiki page. Other information and formatting are provided by the templates. Users need not be aware of the software code in the background. The page is accessible at . Its source code is shown by clicking the 'View Source' tab. 1. Page title: The 12-digit ID describing the structural category of (+)-catechin. 2. Class hierarchy: This information and links are automatically generated from the ID and class information in other Wiki pages. 3. Molecular structure: This picture is generated from the MOL-format file on the server associated with this page. 4. Molecular information: These values are automatically generated from the MOL-format file.

The data page of catechin only includes the page title (12-digit ID), English names, and link information for other databases (Figure 2). The rest of the information and the display style are generated by the template mechanism. The page source is written as a labelled list of data, which is subsequently formatted into a table by the template mechanism. The fundamental difference between the original MediaWiki and our implementation is addressed below.

### Page dependency and dynamic generation of page contents

Page contents are dynamically generated from other page contents through text-string searches at the time of page browsing. The introduction of page dependency is a marked difference from previous Wiki-based systems in which, for example, modifications in one page do not affect other pages. In default MediaWiki, unless specially designed pages are prepared by system administrators, all pages are independent. The following case exemplifies the merits of our page dependency.

#### Case example: realization of metabolite-species table

Metabolite-species information is recorded as a separator-delimited list of metabolite IDs and species names in the reference pages and is visualized as a foldable Wiki-table through the template mechanism. A superficially similar table can be viewed in the pages for compounds and plant species, but these are generated on demand from the information in the reference pages. For example, the list of plant species for a particular compound is obtained by a text-string search for its compound ID on all reference pages. An English name for each metabolite ID is likewise retrieved from the corresponding compound page by a dynamic text-string search.

Therefore, the Wiki-pages for compounds and plant species do not contain information of the table, but only the search commands for generation. An update on a reference page such as adding/removing a metabolite-species relationship will be automatically reflected on the corresponding pages for compounds and plant species. Since information is not duplicated, data management efforts are minimized.

A drawback is the cost of dynamic page generation. Since a single page may contain multiple full-text searches on all other pages, page generation may be slow especially for a summary or statistics page. Currently, our database contains over 30,000 pages (including edit logs), and we conservatively estimate 100,000 pages as the limit to run the database on the current DELL PowerEdge server (Intel Xeon5140 2.33 GHz DualCore, 8 GB memory).

### Introduction of fully dependent pages

As a natural extension of page dependency, we can let the system generate fully dependent pages. Pages for statistics, indexes, or search summaries belong to this category. Traditional Wiki systems do not support such pages unless specially designed as built-in pages because they are devoid of original contents or titles. We prepared two types of fully dependent pages: those that are not necessarily saved, and those that should be saved and subject to page (or internet) searches. Examples of the former are different display styles of an identical page or volatile information that needs not be searched for. Examples of the latter are the index pages. To support both functionalities, the namespace "volatile" and "persist" were newly introduced.

Pages in the *volatile *namespace describe not data contents but function to process data. This resembles, but is clearly different from, the template mechanism. Whereas a template page is called from inside normal Wiki texts and its output is embedded where the template is called, a *volatile *page is linked from other pages with arguments and the processed output is shown as if it were the page contents of the called volatile page. Therefore, the page view may change for each access depending on the given arguments. Its function is the same as the widely used parameter passing mechanism through the http protocol (post/get method in CGI, or common gateway interface), except that any user can design such a page through Wiki.

#### Case example: author summary

From more than 4500 article information, author statistics can be generated: for example the number of compounds reported by the author and the annual publication trend. Since the data are fully dependent on reference pages, the page for the author summary was placed in the *volatile *namespace. It accepts an author's name as a sole argument and generates statistics for the author's publications from all reference pages.

On the other hand, pages in the *persist *namespace initially contain nothing and the page contents are fully projected from other pages. While a page in *volatile *namespace offers function as its page body and receives arguments to be processed, a page in *persist *namespace receives both arguments and function body from other pages. Each time the contents are projected, the corresponding *persist *page retains the projected information so that the data are cashed and subjected to web searches.

#### Case example: molecular indexes

Indexes of molecular names, molecular weights, or chemical formulas are indispensable information for large-scale data collections. Index pages are fully dependent on other pages and should be automatically updated when molecules are added or deleted. Although a list of molecules may be displayed using the #SearchLine function (see Methods section) in *volatile *namespace, such a list cannot be utilized for further full-text searches because the list is not materialized as the page body. Therefore, the *persist *namespace is required to realize such fully dependent pages whose page sources list data rather than functions to generate lists. In our implementation, indexes of the exact mass, average mass, molecular name, and chemical formulas were prepared in the *persist *namespace.

## Discussion

### Search commands alleviate administrators' load

Traditional HTML-based systems including Wiki provide a collection of independent pages only. Therefore, it is difficult to obtain page summaries or identify discrepancies on pages even inside the same website. To acquire statistics, for example, users must rely on search engines, or administrators must prepare CGI-based functions to provide user-dependent views.

The installation of embeddable query commands alleviates, at least in part, these difficulties. It yields (fully) dependent pages; page statistics or indexes can be easily designed and maintained, provided that data pages are written in a uniform format. On Wiki-based systems, moreover, not only administrators but also users have the right to create such pages.

The introduction of embedded queries thus changes the way Wiki-based systems are used. It accelerates the exchange of information and reduces the cost of maintaining data integrity through appropriately designed, dependent pages. However, creating dependent pages requires understanding of MediaWiki system and additional programming skill (interested readers are encouraged to view page sources of our website). Thus, our contribution rather benefits advanced users that can write dependent (i.e. programmed) pages than the majority of end users.

### Related Wiki-based systems

Our implementation on MediaWiki for flavonoid data is a novel, though small-sized, prototype for *interdependent *web pages. There have been attempts to utilize Wiki-based systems for gene annotation or for the management of biological information [12-14]. However, embeddable queries are not supported, rendering it difficult to provide statistics or summary pages. A notable exception is BOWiki, which supports a reasoning engine for description logic and can introduce *n*-nary relationships among pages to manage ontology data [15]. In terms of search function, most Wiki-based systems either use the build-in, simple text search command, or install Google extension as in WikiPathways [16]. In both cases, search results are only listed in a custom page. To the authors' knowledge, no Wiki-based systems have supported embeddable search commands. As the scripting language, we chose Lua for its lightweight and simple design as an embeddable language.

### Advances from MediaWiki

MediaWiki supports streamlined page formatting through the so-called template mechanism. A tabulated list of data can be transformed into a uniform style by using this mechanism. Our implementation also extensively utilizes the function, but we additionally exploit the #repeat command so that the system can handle an arbitrary number of data records (see Methods section). In the original MediaWiki, only a fixed number of arguments are allowed for each template (or command) and there is no mechanism to handle data of unknown size. Under this restriction, processing search results is impossible because the number of returned items is unpredictable. Thus, the introduction of loop- or control structures is the fundamental difference from MediaWiki and other Wiki-based systems. In other words, we allow users to write small computer programs inside Wiki pages, i.e., to realize document computing.

### Placement of dependent information

The existence of dependent pages raises the problem of data placement. In general, when page contents are generated from original data applied to a function, the result can be placed at the location of (1) the original data, (2) the function, or (3) elsewhere (Table 1): Case (1) is the template function on MediaWiki; a function in the *template *namespace is called by the original data and the result is embedded where the template name appears. Case (2) is a page in the *volatile *namespace; it resembles the http-based parameter passing in CGI in which users input arguments to show user-dependent views. Case (3) is a page in the *persist *namespace; it resembles the Resource description framework Site Summary (RSS) concept in which data-crawling software, called a feed reader, scans registered news-sites or web logs to obtain real-time news. Retrieved information may be cast as an independent web page elsewhere. A well-known example is Google News [17]. The difference from RSS is that a *persist *page is updated at the time of browsing, and its contents persist as if it were an independent page until the next update.

**Table 1:** Data placement policy of Wiki pages

**Page type**	**Contents**	**Required data**	**Data placement**	**Similar web facility**
Normal	data	N/A	this page	HTML
*Template*	function body	function arguments	function caller	MediaWiki
*Volatile*	function body	function arguments	this page	CGI*
*Persist*	N/A	function body & function arguments	this page	RSS

(* ... http-based parameter passing; CGI ... common gateway interface; RSS ... resource description framework (RDF) site summary)

### Security issues

The embedding of the Lua programming language on MediaWiki made it possible to implement any program code (see Methods section). It was too powerful a function to suppress server attacks. To avoid denial-of-service (DOS) or other attacks through Lua, we closed its I/O function and restricted its running time to 10 seconds. A page-size quota was also set. Therefore, it became impossible to handle truly large-scale data (e.g. one million pages) on our system. However, this limitation is due to our system maintenance and is not a theoretical limitation (provided that we can use a high-performance computer as our server).

Since all pages are accessed through the http protocol, all pages are subject to web vandalism. Although the original MediaWiki supports a password-based edit restriction, this does not work for *volatile *and *persist *pages, which are updated only by visiting these pages. Although both types are fully dependent and can be easily reconstructed, vandalized *persist *pages may be cached by external web search engines (e.g. Google). To prevent this, in our current implementation all pages are password-protected, and *persist *pages are updated only through accesses by registered users.

## Conclusion

We proposed the introduction of embedded string-search commands on MediaWiki, and realized a half-formatted database system that can handle relational operations. The introduction of embedded string-searches created the notion of page dependency and document computing. Dependent pages are categorized into three types, each corresponding to standard Wiki pages, http-based parameter passing in CGI, and RSS-like pages. Thus, page dependency sorts out seemingly irrelevant web functionalities in a streamlined manner. The advantage is that any (registered) user can create such pages through a web browser.

Using these functionalities, a database of around 7,000 flavonoid molecules was implemented. On our system, the task of data input and maintenance was rendered easier and systematic queries became possible through a tabulated data format.

The introduction of embeddable search commands is theoretically possible for standard HTML with Java script. If searching against external servers were to be realized, the concept will change how we manage information on the web. Imagine a world where every user obtains a power to design and construct statistics pages as is done by Google: a world where construction of 'meta' knowledge becomes much easier. To accelerate information exchanges and to improve data integrity on the web, what is anticipated is a framework like our system for the entire internet.

## Competing interests

The authors declare that they have no competing interests.

## Authors' contributions

MA incepted and conducted the research and wrote the manuscript. KS helped with implementation. Both authors read and approved the final manuscript.
